# Vaccines and immunotherapy against fungi: the new frontier

**DOI:** 10.3389/fmicb.2013.00006

**Published:** 2013-01-25

**Authors:** Joshua D. Nosanchuk, Carlos P. Taborda

**Affiliations:** ^1^Department of Medicine, Albert Einstein College of MedicineBronx, NY, USA; ^2^Department of Microbiology and Immunology, Albert Einstein College of MedicineBronx, NY, USA; ^3^Laboratory of Medical Mycology-LIM53/IMTSP, Department of Microbiology, Institute of Biomedical Sciences, University of São PauloSão Paulo, Brazil

Invasive fungal diseases have increased many fold over the past 50 years. Current treatment regimens typically require prolonged administration of antifungal medications that can have significant toxicity. Moreover, our present potent antifungal armamentarium fails to eradicate fungal pathogens from certain compromised hosts. Additionally, invasive fungal diseases continue to have unacceptably high mortality rates. A growing body of work has focused on the utility of vaccines and/or immunotherapy as a powerful tool in combating mycoses, either for the active treatment, as an adjuvant, or in the prevention of specific fungal pathogens. This Research Topic “Vaccines and Immunotherapy against fungi: a new frontier” in *Frontiers in Fungi and their Interactions* details the exciting progress in developing vaccines and immunotherapy for fungi.

The critical requirement for understanding the degrees of engagement of host defense pathways in responding to fungal invasion has led to an increased focus on host-pathogen interactions. In this Research Topic, Carvalho et al. (2012) review our current progress on this endeavor and underscore the need for coordinated cross-disciplinary future efforts. A major focus of the special Topic is the advance of vaccine strategies against major fungal pathogens. To this extent, the issue focuses on developments in vaccine strategies against *Candida albicans* (Vecchiarelli et al., 2012), *Aspergillus fumigatus* (Diaz-Arevalo et al., 2012), *Cryptococcus neoformans* (Hole and Wormley, 2012), and *Paracoccidioides brasiliensis* (Travassos and Taborda, 2012). Progress in optimizing adjuvants for a vaccine against *P. brasiliensis* is also presented (Mayorga et al., 2012). Shifting host responses to facilitate fungal clearance is shown in work utilizing ArtinM, a D-mannose binding lectin from *Artocarpus heterophyllus*, which modulates immunity against *P. brasiliensis* (Ruas et al., 2012). Along this line, information is presented regarding the immunomodulatory effects of fungal immunogens and how they impact disease (Rodrigues and Nimrichter, 2012). The utility of antibody based therapeutic approaches is presented against a specific fungus, *Histoplasma capsulatum* (Nosanchuk et al., 2012), and as a broad-spectrum therapeutic using antibody labeled with fungicidal nuclides (Nosanchuk and Dadachova, 2012). A therapeutic monoclonal antibody in early phase research for sporotrichosis is also detailed (Almeida, 2012). Moreover, the broad potential of antibody-derived “killer peptides” is presented (Magliani et al., 2012). Finally, new information about the antifungal activity of “old” drugs is discussed. Hydroxyurea is shown to impact the sphingolipid pathway underscoring the role of these compounds in fungal biology (Tripathi et al., 2012). The diverse activities of Amphotericin B on both fungi, impacting ergosterol stability and cellular morphology, and host cells, engaging pattern receptors, is critically summarized (Mesa-Arango et al., 2012).

In sum, these articles broadly paint the current spectrum of investigations on host-pathogen interactions and provide a review of the state-of-the-art in vaccinology and immunotherapy against fungi. The information presented also underscores the rich areas for future study, all promising improved therapeutics against fungal invaders.
